# Three-dimensional evaluation of stem placement accuracy with the conventional guide in reverse shoulder arthroplasty and its relevance to clinical outcomes

**DOI:** 10.1016/j.jseint.2024.09.030

**Published:** 2024-11-08

**Authors:** Katsumasa Nakazawa, Tomoya Manaka, Yukihide Minoda, Yoshihiro Hirakawa, Yoichi Ito, Hayato Shimizu, Ryosuke Iio, Hiroaki Nakamura

**Affiliations:** a Department of Orthopaedic Surgery, Osaka Metropolitan University Graduate School of Medicine, Osaka, Japan; b Department of Orthopaedic Surgery, Ishikiriseiki Hospital, Higashiosaka, Japan; c Osaka Shoulder Center, Ito Clinic, Osaka, Japan; d Shimizu Orthopaedic Clinic, Osaka, Japan

**Keywords:** Stem placement, Retroversion of the stem, Reverse shoulder arthroplasty, Clinical outcomes, 3D postoperative evaluation system, Range of motion

## Abstract

**Background:**

Placement of retroversion of the stem (RS) is important in reverse shoulder arthroplasty. A conventional guide, based on the forearm, has been used for stem placement; however, only a few studies have reported the accuracy of stem placement using conventional guides. In this study, a three-dimensional postoperative evaluation software was used to investigate the accuracy of RS placement using a conventional guide and its effect on postoperative outcomes.

**Methods:**

This retrospective study was performed by a single surgeon (a board-certified specialist with more than 15 years of experience in performing reverse shoulder arthroplasty) using the Exactech Equinoxe Reverse Shoulder System (Exactech Inc., Gainesville, FL, USA). Forty-nine patients who were followed up for at least 2 years were included. The target RS angle of the humeral component was set to 20°. Postoperative implant placement, including RS, was assessed with a three-dimensional planning software using computed tomography images obtained postoperatively. Postoperative range of motion and its relationship with clinical outcomes were also evaluated as clinical assessment. Furthermore, a subanalysis was performed comparing the 0-20° RS group with the other groups.

**Results:**

The mean postoperative RS was 13.2 ± 11.9° and was placed within 0-20° in 31/49 patients (63.3%). A correlation was observed between postoperative external rotation and RS (r = 0.30, *P* = .03). In a further subanalysis, the Constant–Murley score was significantly higher in the 0-20° RS group (*P* = .03).

**Conclusion:**

Placement of the RS using a conventional guide varied from the target position. RS correlated with postoperative external rotation, and RS within 0-20° significantly improved clinical outcomes. These results suggested that accurate placement of the RS may improve clinical outcomes. Therefore, the development of surgical assistive technologies for accurate placement is necessary to ensure accurate stem placement to avoid human error.

Reverse shoulder arthroplasty (RSA) is an effective treatment for patients with rotator cuff tear arthropathy, rheumatoid arthritis, failed conventional anatomic shoulder arthroplasty, or proximal humeral tumors.^1^^,^^2^^,^^23^ However, the ideal implant placement remains controversial. The position of the glenoid component is particularly important, and its incorrect placement may lead to instability, scapular notching, base acromion fractures, and catastrophic failures in RSA.^9^^,^^16^^,^^27^^,^^34^ Therefore, surgical assistive technologies such as navigation and patient-specific implants have been introduced in recent years and have reported good implant placement and clinical outcomes.^14^^,^^15^^,^^31^ In contrast, regarding the position of the humeral component, Stephenson et al reported a decreased range of external rotation (ER) in anteversion placement in a biomechanical study, and a biomechanical study by Gulotta et al reported an increase in ER with increased retroversion and in internal rotation (IR) range of motion (ROM) when placed within 0-20°.^10^^,^^29^ Rhee et al also reported no significant difference in the ROM between 0° and 20° retroversion placement.^26^ However, ideal retroversion placement remains controversial. In addition, conventional guide placement based on the forearm is commonly used; however, the accuracy of these placement positions has been reported less frequently.

Therefore, this study aimed to assess the placement status of implants and accuracy of the placement using the ZedShoulder software (Lexi, Tokyo, Japan), a three-dimensional (3D) postoperative evaluation system that can automatically measure the postoperative retroversion of the stem (RS)^3^^,^^23^ and to evaluate the impact of implant placement on clinical outcomes.

We hypothesized that the placement accuracy of stems using a conventional guide is low, and that the clinical outcome improves in cases where the RS is placed at 0°-20°.

## Materials and methods

### Patients

This was a retrospective study; we included 83 patients who underwent RSA using the Exactech Equinoxe Reverse Shoulder System (Exactech Inc., Gainesville, FL, USA) among the 137 patients who underwent RSA between August 2017 and June 2021 at a single institution.

The inclusion criteria were patients who had undergone RSA for cuff tear arthropathy or irreparable rotator cuff tear and were able to be followed up for at least 2 years after surgery. The exclusion criteria were revision RSA, fracture sequelae, proximal humeral fracture, dislocation or infection, and acute proximal humeral fracture.

Among the 83 patients, we excluded 4 revision cases, 10 fracture sequelae cases, 4 proximal humeral fracture cases, 3 infection cases, and 5 dislocation cases.

Eventually, 8 patients were lost to follow-up and 49 patients (33 men, 16 women) were followed up for more than 2 years after surgery. The mean follow-up rate was 86.0%. Informed consent was obtained from each patient and ethical approval was obtained from the institutional review board.

### Surgical procedure

All procedures were performed by a single surgeon (T.M.) using a single implant (Exactech Equinoxe Reverse Shoulder System; Exactech Inc., Gainesville, FL, USA). The surgeon was a board-certified specialist with more than 15 years of experience. All procedures were performed using the standard deltopectoral approach.

For the humeral-side operation, the humerus was osteotomized at an angle of 132.5° at the anatomical neck using an extramedullary guide, the Fixed Angle (132.5°) Osteotomy Guide, and set at a retroversion angle of 20° to the forearm. The Fixed Angle Osteotomy Guide was fixed with a K-wire. Subsequently, the humeral shaft was reamed using a broach; during broaching, a retroversion rod was attached to the broach handle and inserted 20° relative to the forearm. Next, the stem was inserted. For the stem insertion, the retroversion rod was also attached to the stem inserter and inserted 20° to the forearm.

The baseplate was fixed to the glenoid using a central screw and three to four peripheral screws. The subscapularis muscle was repaired to the extent possible and was treated by peel dissection from the attachment of the lesser tuberosity. Before the placement of humeral component, three high-strength sutures (No. 2) were looped through the proximal humerus, followed by three sutures through the subscapularis muscle and sutured with a Nice knot. ^7^

All patients underwent the same postoperative protocol and used a sling for 2 weeks. Assisted ROM exercises were started 2 days postoperatively, and the free ROM strengthening program began 3 months after surgery.

### Computed tomography image analysis and virtual arthroplasty

**Figure 1:**
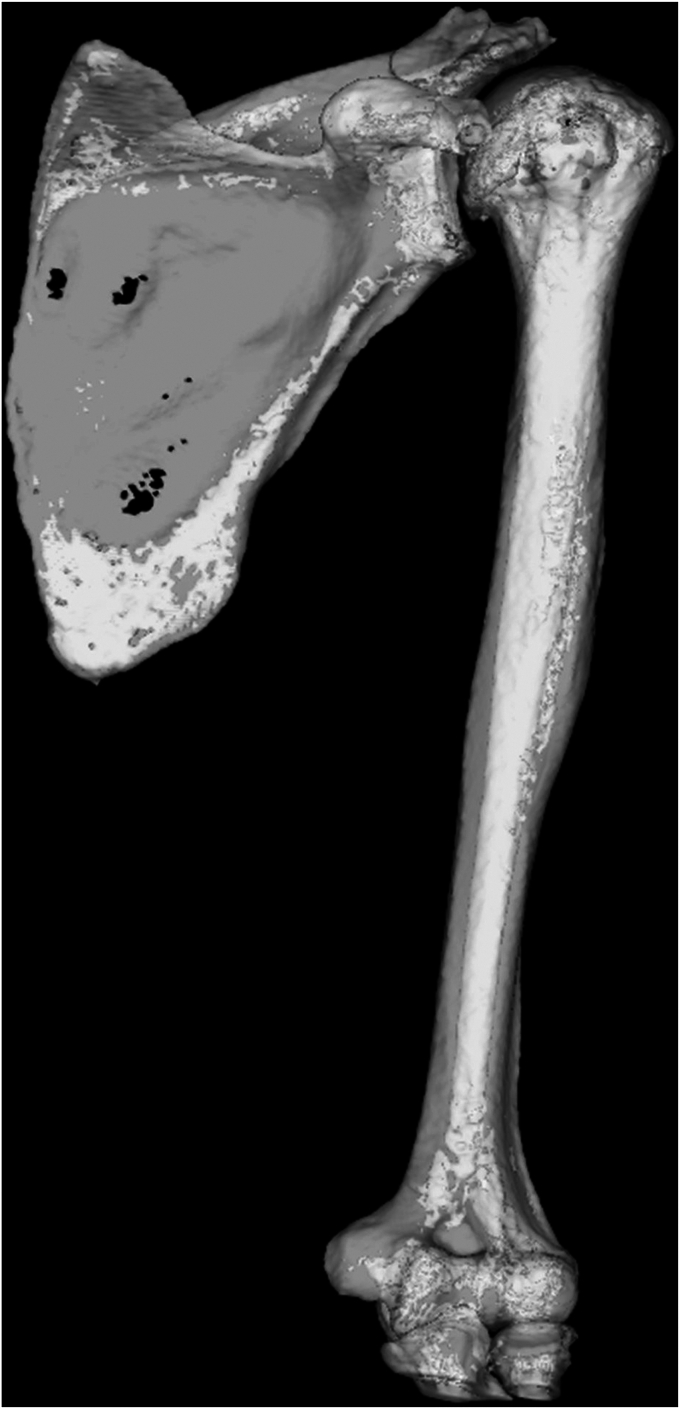
3D bone model created from preoperative computed tomography data of the scapula.

Computed tomography (CT) was performed preoperatively and at 1 month postoperatively with a Digital Imaging and Communications in Medicine format and 1 mm slice of the scapula and humerus as the imaging condition. ZedShoulder software (Lexi) was used for the measurements. The software selected reference points based on preoperative CT data of the scapula and humerus and created a semiautomated 3D bone model (Fig. 1).^23^

**Figure 2:**
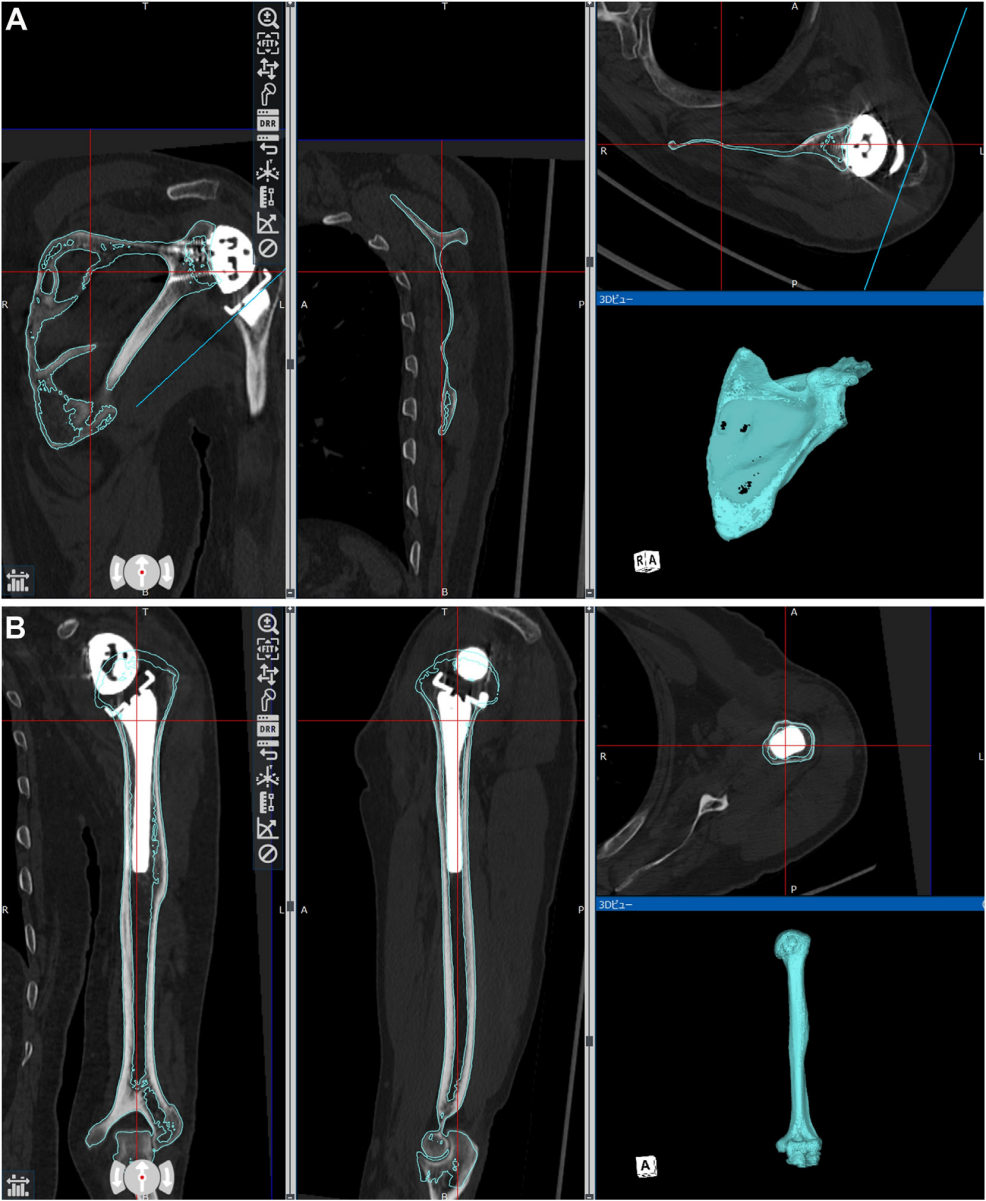
Method of superimposing postoperative computed tomography and preoperative data. **A**: Matching with the scapula. **B**: Matching with the humerus.

**Figure 3:**
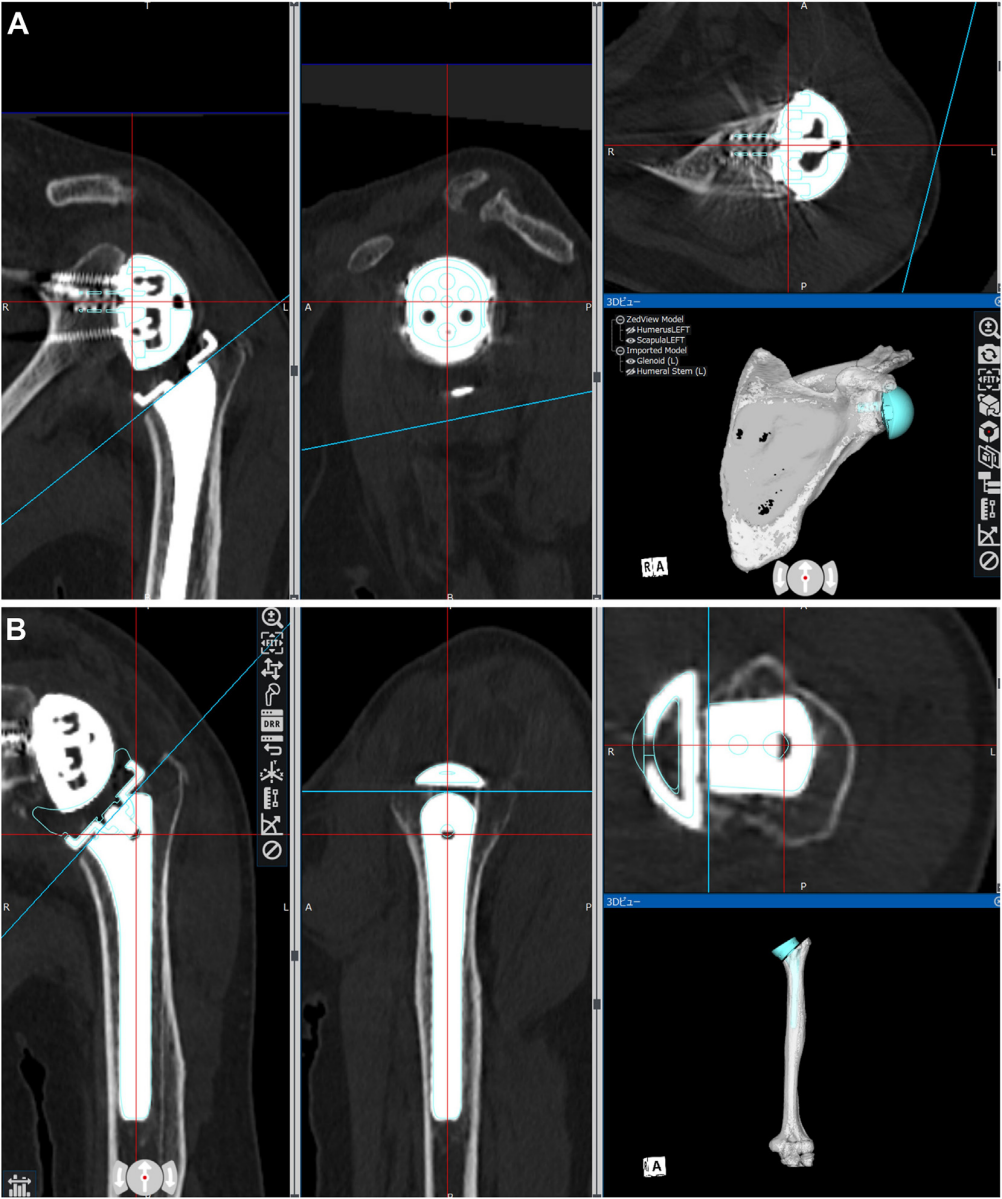
Method of matching implants. Computer-aided design (CAD) models of the Exactech Equinoxe Reverse Shoulder System for the implants in postoperative computed tomography data. **A**: Matching with the scapular component. **B**: Matching with the humeral component.

**Figure 4:**
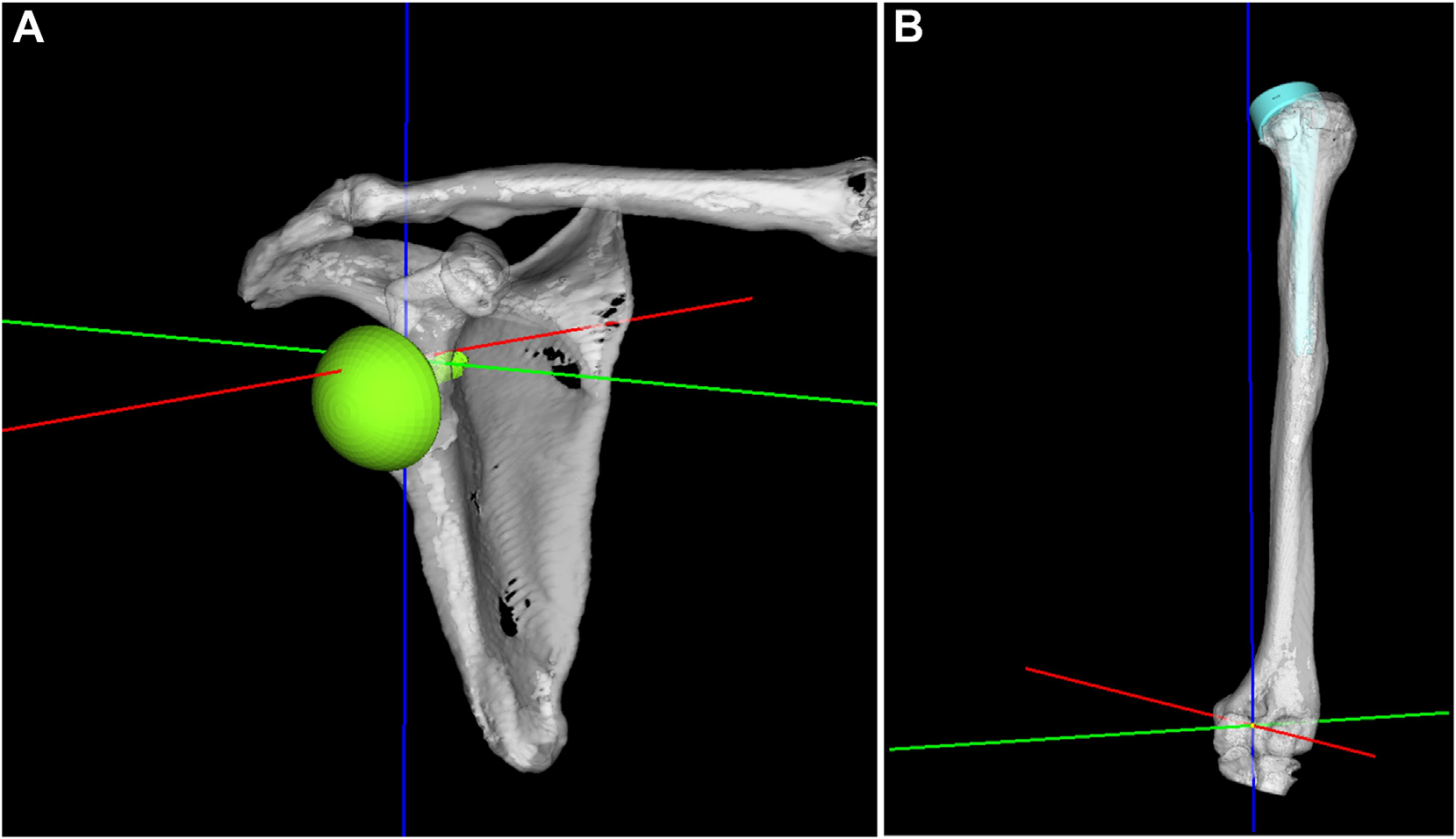
Coordinate system. **(A)** Scapular plane was created connecting the midpoint of the glenoid width, the medial angle of the scapula, and the inferior angle of the scapula, with the X-axis () as the midpoint of the medial angle of the scapula and the glenoid width, the Z-axis () as the point perpendicular to the X-axis and the Z-axis on the plane, and the Y-axis () as the point perpendicular to the X and Z axes. **(B)** The humerus coordinate system was created by creating a plane with three points: the lesser tubercle of the humerus, anterior trochlea of the humerus, and anterior capitulum of the humerus. The line connecting the anterior trochlea of the humerus and anterior capitulum of the humerus was the X-axis, the intersection of the line drawn from the lesser tubercle of the humerus to the X-axis was the origin, the origin and lesser tubercle of the humerus were the Z-axis, and the axis orthogonal to the X- and Z axes was the Y-axis.

A 3D model was then created from the postoperative CT images and superimposed on the preoperative data (Fig. 2, *A* and *B*). Next, the computer-aided design models of the Exactech Equinoxe Reverse Shoulder System were manually matched to the implant sites in the postoperative CT data and evaluated (Fig. 3, *A* and *B*). The scapula co-ordinate system is a plane connecting the midpoint of the glenoid width, medial border of the scapula, and inferior angle of scapular, with the X-axis as the midpoint between the medial border of the scapula and glenoid width, Z-axis as the point perpendicular to the X-axis, and Y-axis as the point perpendicular to the X and Z axes (Fig. 4, *A*).

The humerus co-ordinate system was created by creating a plane with three points: the lesser tubercle of the humerus, anterior trochlea of the humerus, and anterior capitulum of the humerus. The line connecting the anterior trochlea of the humerus and anterior capitulum of the humerus was the X-axis; the intersection of the line drawn from the lesser tubercle of the humerus to the X-axis was the origin; the origin and lesser tubercle of the humerus were the Z-axis; and the axis orthogonal to the X- and Z-axes was the Y-axis (Fig. 4, *B*).

The distance from the center of the glenoid fossa to the outermost point of the greater tuberosity of the humerus was defined as the global offset (GO) and measured.

The glenoid version (GV) was measured from the angle between the line connecting the medial border of the scapula and anteroposterior midpoint of the baseplate and the line perpendicular to the baseplate.

The glenoid inclination (GI) was measured as the angle between the line connecting the medial border of the scapula and upper and lower midpoints of the baseplate and the line perpendicular to the baseplate. RS was measured with respect to the medial and lateral humeral epicondyles. Preoperative GV, GI, humeral retroversion (HR), and postoperative RS, GO, GV, and GI were automatically calculated. Postoperative measurements were performed by a single assessor, who was an orthopedic surgeon.

### Clinical outcomes

All patients underwent standardized clinical and radiological examinations by an examiner other than the primary surgeon at 1 and 2 years postoperatively. In this study, the ROM (flexion, abduction, ER, and IR) and Constant–Murley score^8^ at 2 years postoperatively, American Shoulder and Elbow Surgeons (ASES) score, and pain visual analog scale (VAS) were measured on a scale of 0 (no pain) to 10 (worst pain). ROM was measured in flexion and abduction and ER using a handheld goniometer. IR was measured as the vertebral level reached by the tip of the thumb in a seated position and scored according to previous reports: 1-12 if the 1-12 thoracic vertebrae were reached; 13-17 if the 1-5 lumbar spine were reached; and 18 if the sacrum or region below the sacrum was reached.^6^^,^^24^

To assess the association with RS, patients with an RS within 0-20° were categorized into group 1, and the other patients were categorized into group 2. A subanalysis was performed using these groups.

### Statistical analysis

The Shapiro–Wilk test was used to test for normality. Data analysis included descriptive statistics such as mean, standard deviation, and 95% confidence intervals. Intraclass correlation coefficients (ICCs) were calculated using standard statistical methods (ICC 1, 1; intraobserver reliability). The ICCs for intraobserver reliability were calculated using data from postoperative GV, GI, GO, and RS.

Pearson’s correlation coefficients were used for comparison between postoperative GV, GI, GO, and RS and outcomes. Student’s t-test or the Mann–Whitney U-test was used for group comparisons. The Fisher's direct probability test was used for discrete variables in group comparisons.

The level of signiﬁcance was set at *P* < .05. Statistical analyses were performed using SPSS software (version 25.0; IBM Corp., Armonk, NY, USA), and G Power software (version 3.1.9; Heinrich Heine Universität, Düsseldorf, Germany) was used to perform post hoc power analyses.

## Results

### Patient demographics

The preoperative patient demographics are listed in Table I. The patients’ average age was 76.6 ± 6.8 years (range, 65-90 years); 28 patients had surgery on the right side and 21 had surgery on the left. The average height, weight, and body mass index were 157.8 ± 9.4 cm (136.2-171.5), 59.7 ± 10.6 kg (38-85), 23.9 ± 3.5 kg/m^2^ (17.3-33.6), respectively. This study included 7 patients with diabetes and 30 patients with hypertension or heart disease.

**Table I:** Patient background.

Characteristic	
Goutalier classification	
Fatty infltration of the supraspinatus	3.1 ± 0.8
Fatty infltration of the infraspinatus	2.9 ± 1.2
Fatty infltration of the subscapularis	2.5 ± 1.2
Fatty infltration of the teres minor	1.1 ± 1.2
Classification of massive rotator cuff tear arthritis and glenoid morphology	
Hamada classification	Grade 2, 4; Grade, 7; Grade 4A, 17; Grade 4B, 19; Grade 5, 2
Walch classification	A1, 45; A2, 4
Favard classificaion	E0, 42; E1, 3; E2, 4
3 dimensional preoperative measurement	
Humeral retroversion (°)	36.3 ± 11.6 (8.1-55.6)
Glenoid version (°)	1.0 ± 8.9 (−13.8 to 41.9)
Glenoid inclination (°)	4.1 ± 9.2 (−30.1 to 27.9)
Subscapularis repair	Yes, 45; no, 4
Navigation	Yes, 29; no, 20
Cemented	Cementless, 49
Tendon transfer	Yes, 49; no, 0
Glenosphere diameter	36 mm, 9; 38 mm, 30; 42 mm, 10
Augmented baseplate use	Yes, 19; no, 30
Number of baseplate screws	3.6 ± 0.5
Preopearative clinical outcome	
Flexion (°)	67 ± 37 (10-150)
Abduction (°)	62 ± 31 (15-145)
External rotaion (°)	23 ± 18 (−10 to 60)
Internal rotation (points)	15.3 ± 3.6 (7-18)
Constant-Murley score	30.1 ± 14.4 (8-67)
ASES score	42.0 ± 24.8 (0-95)
VAS	5.0 ± 3.4 (0-10)

*ASES*, American Shoulder and Elbow Surgeons; *VAS*, visual analog scale.

Data are presented as mean ± standard deviation.

Regarding shoulder usage, all patients showed a low level of daily life only; 30 patients needed surgery in their dominant hand and 19 in their nondominant hand. According to the Hamada classification, 4 patients were classified as grade 2, 7 patients as grade 3, 17 patients as grade 4A, 19 as grade 4B, and 2 as grade 5.^12^ According to the Walch classification, 45 patients were classified as type A1 and 4 as type A2.^32^ According to the Favard classification, 42 patients were classified as E0, 3 as E1, and 4 as E2.^28^ In addition, 3D preoperative measurements showed a mean GV of 1.0° ± 8.9° and GI of 4.1° ± 9.2°, with a mean HR of 36.3° ± 11.6°.

Subscapularis repair was performed in 45 patients; navigation was used in 29 patients, and cementless stem was used in all patients. Normal type polyethylene inserts (+0 mm) were used in all cases.

The mean follow-up period was 35.7 ± 11.8 months (24-72). Postoperative complications included postoperative dislocation in two patients; however, this did not progress to frequent dislocation. Therefore, the patients were treated conservatively. No apparent infections or acromial fractures were observed.

### Postoperative 3D measurement

The intraobserver ICCs for postoperative parameters were 0.97 (0.92-0.99), 0.98 (0.97-0.99), 0.96 (0.91-0.98), and 0.86 (0.71-0.94) for RS, GV, and GI, respectively. Postoperative 3D measurements are listed in Table II. The mean GO was 51.5 ± 5.1 mm, GV was 1.5° ± 6.2°, and GI was −0.5° ± 5.4°.

**Table II:** Postoperative 3D parameters and postoperative clinical outcomes.

Characteristic	
Preoperative 3dimentional measurement	
Global offset (mm)	51.5 ± 5.1 (33.7-61.2)
Glenoid version (°) Anteversion; + retroversion; −	1.5 ± 6.2 (−12.8 to 20.0)
Glenoid inclination (°) Superior; + Inferior; −	−0.5 ± 5.4 (−11.3 to 15.0)
Retroversion of humerus (°)	13.2 ± 11.9 (−12.6 to 38.0)
Postoperative outcome	
Flexion (°)	114 ± 24 (70-170)
Abduction (°)	102 ± 25 (60-170)
External rotaion (°)	28 ± 21 (−30 to 70)
Internal rotation (points)	16.4 ± 2.6 (6-18)
Constant-Murley score	61.0 ± 13.6 (25-83)
ASES score	67.0 ± 20.7 (10-100)
VAS	2.4 ± 2.5 (0-8)

*ASES*, American Shoulder and Elbow Surgeons; *VAS*, visual analog scale.

Data are presented as mean ± standard deviation.

**Figure 5:**
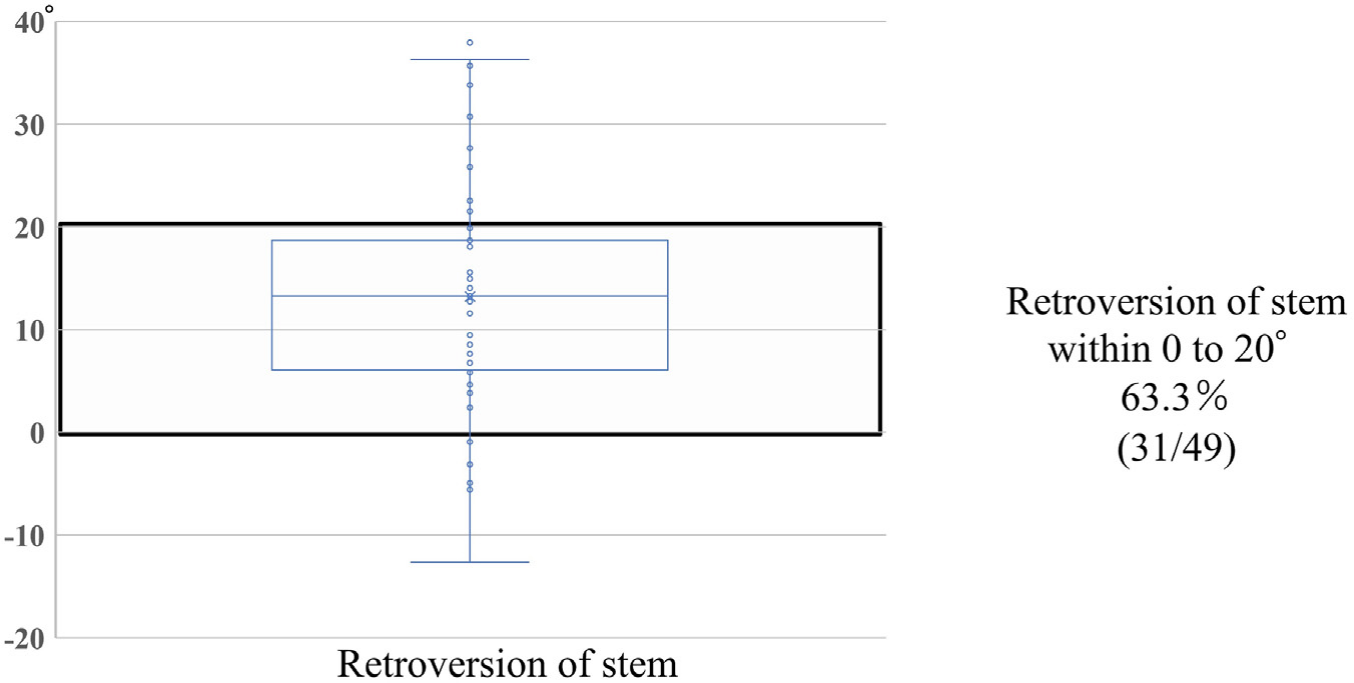
Box-and-whisker diagram of retroversion of stem placement. X shows the mean value, ○ is the plot; 31/49 cases were placed within 0-20°, and 7 cases were placed below 0°.

The mean RS was 13.2° ± 11.9°, and RS was placed within 20° of 0° in 31/49 patients (63.3%). In addition, RS was placed in anteversion in seven patients (14.2%). The average error from the installation target was −6.8° ± 11.9° (Fig. 5).

### Correlation with clinical outcome

**Figure 6:**
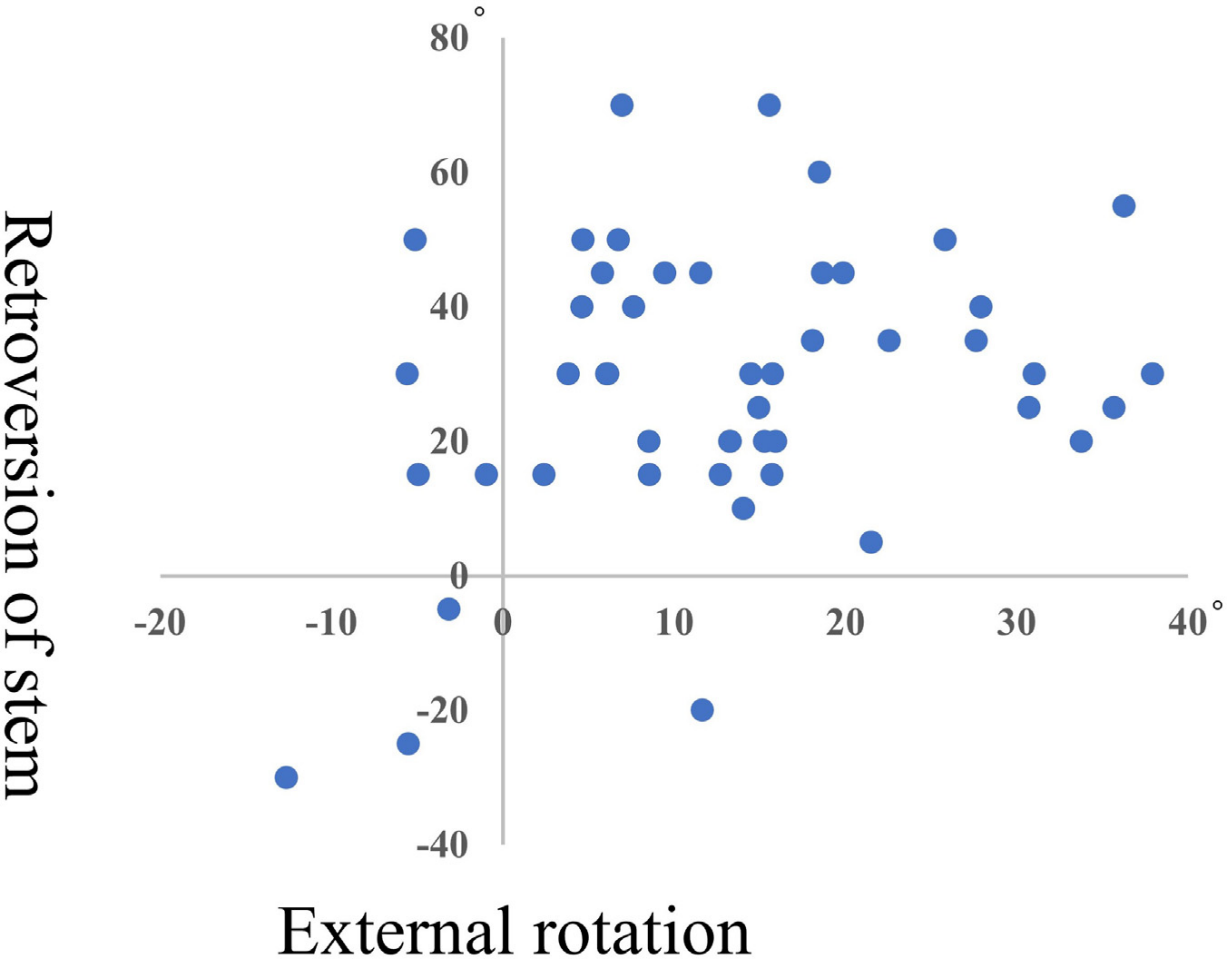
Correlation diagram between retroversion of stem and external rotation.

The postoperative clinical outcomes are listed in Table II. We observed a correlation between RS and postoperative ER (r = 0.30, *P* = .03) (Table III, Fig. 6). The post hoc power analysis indicated that the statistical power of the correlation test was 0.60, indicating a moderate ability to detect the observed effect size.

**Table III:** Implant position in relation to postoperative clinical outcomes.

	Flexion	Abduction	External rotation	Internal rotation	Constant-Murley score	ASES score	VAS
Global offset							
Correlation coefficient (p)	0.05	0.23	0.11	−0.16	0.21	0.19	−0.15
*P* value	.74	.12	.44	.29	.14	.20	.31
Glenoid version							
Correlation coefficient (p)	0.05	0.08	0.01	−0.08	0.06	0.04	−0.07
*P* value	.71	.57	.95	.62	.67	.79	.64
Glenoid inlcination							
Correlation coefficient (p)	−0.18	−0.27	−0.10	−0.05	−0.13	−0.20	0.19
*P* value	.23	.07	.50	.76	.36	.16	.19
Retroversion of stem							
Correlation coefficient (p)	0.06	−0.60	0.30	−0.66	−0.05	−0.02	0.08
*P* value	.71	.68	.03∗	.66	.74	.90	.60

*ASES*, American Shoulder and Elbow Surgeons; *VAS*, visual analog scale.

∗*P* < .05.

### Comparison between groups 1 and 2

There were no significant differences in patient backgrounds between the groups (Table IV). In addition, no significant differences in the postoperative GI were observed; however, the GI was inferior in group 2. Postoperative clinical outcomes were significantly better in group 1 according to the Constant–Murley score (*P* = .03) (Table V). However, no significant difference was observed in postoperative ER, although it was greater in group 1 (*P* = .07).

**Table IV:** Patient background for group 1 (retroversion of stem within 0-20°) and group 2 (other groups).

	Group 1	Group 2	*P*∗
Age (y)	76.5 ± 7.7	76.7 ± 5.1	.90
Sex (male: female)	22: 9	11: 7	.48
Dominant side surgery	18: 13	12: 6	.55
BMI (kg/m^2^)	23.8 ± 3.1	24.1 ± 4.2	.80
Preoperative 3D measurement			
preoperative glenoid version (°)	1.6 ± 10.3	−0.2 ± 6.3	.50
preoperative glenoid inclination (°)	2.6 ± 10.0	6.6 ± 7.4	.15
Humeral retroversion (°)	35.6 ± 12.1	37.5 ± 10.9	.59
Subscapularis repair (repaired: not repaired)	27: 4	18: 0	.11
Navigation (navigation: nonnavigation)	17: 14	12: 6	.42
Augmented baseplate used (augmented baseplate: Standard baseplate)	11: 20	8: 10	.53
Glenosphere diameter			
36 mm	6	3	
38 mm	20	10	
42 mm	5	5	.95
Goutalier classification			
Fatty infltration of the supraspinatus	3.0 ± 0.9	3.2 ± 0.7	.38
Fatty infltration of the infraspinatus	2.9 ± 1.2	2.9 ± 1.2	.98
Fatty infltration of the subscapularis	2.5 ± 1.2	2.5 ± 1.2	.93
Fatty infltration of the teres minor	1.0 ± 1.1	1.2 ± 1.3	.63
Preoperative outcome			
Flexion (°)	67 ± 39	67 ± 35	.99
Abduction (°)	63 ± 33	59 ± 28	.69
External rotation (°)	22 ± 18	24 ± 20	.80
Internal rotation (points)	16.6 ± 2.7	16.1 ± 2.5	.53
Constant-Murley score	26.2 ± 14.8	32.4 ± 13.9	.17
ASES score	36.2 ± 26.2	45.3 ± 23.8	.23
VAS	6.1 ± 3.0	4.3 ± 3.5	.08
Postoperative 3D measurement			
Postoperative glenoid version (°)	2.4 ± 6.0	−0.1 ± 6.5	.19
Postoperative glenoid inclination (°)	1.6 ± 6.6	−1.8 ± 4.2	.06
Global offset (mm)	52.2 ± 5.3	50.3 ± 4.7	.21
Retrovesion of stem (°)	11.5 ± 5.0	16.3 ± 18.4	.17

*3D*, three dimensional; *ASES*, American Shoulder and Elbow Surgeons; *BMI*, body mass index; *VAS*, visual analog scale.

Data are presented as mean ± standard deviation.

∗ *P* < .05 the statistical analysis was performed using the Mann Whitney U-test, or otherwise using the student's t-test. Fisher's direct probability test was used for discrete variables for group comparisons.

**Table V:** Comparison of postoperative clinical outcomes between group 1 and group 2.

	Group 1	Group 2	*P*
Postoperative outcome			
Flexion (°)	114 ± 22	114 ± 27	.99
Abduction (°)	102 ± 34	99 ± 27	.63
External rotation (°)	32 ± 19	22 ± 24	.07
Internal rotation (°)	16.6 ± 2.7	16.1 ± 2.5	.53
Constant-Murley score	64.1 ± 12.4	55.7 ± 14.2	.03∗
ASES score	69.6 ± 20.9	62.4 ± 20.3	.25
VAS	2.6 ± 2.7	2.1 ± 2.4	.83

*ASES*, American Shoulder and Elbow Surgeons; *VAS*, visual analog scale.

Data are presented as mean ± standard deviation.

∗ *P* < .05 the statistical analysis was performed using the Mann Whitney U-test, or otherwise using the student's t-test.

## Discussion

This study aimed to assess the placement status of implants and accuracy of the placement using a 3D postoperative evaluation system and evaluate the impact of implant placement on clinical outcomes. Subsequently, we observed that on average, the placement of the RS deviated by approximately −6.8° ± 11.9° from the target, and 63.3% of all RS were placed within 0-20°, indicating inaccuracy of placement by the conventional guide. Regarding the relationship between implant placement position and clinical outcomes, the larger the RS, the greater the postoperative ER, and the Constant–Murley score improved with placement between 0° and 20°.

Similar with our findings, a previous report using a conventional guide with a target value of 25° retroversion obtained a mean RS of 21.7° ± 11.9°, suggesting that the RS was incorrectly positioned.^17^ In particular, 14.2% (7/49) of patients in the present study underwent anteversion placement. The anteversion position may restrict the ER outside of adduction and cause posterior instability owing to reduced stability in the posterior direction.^9^^,^^19^ As the placement of the stem using a conventional guide is based on the forearm, errors may occur owing to differences in the holding angle caused by IR and ER stress during the retention of the elbow joint and morphological characteristics of the forearm. In addition, postoperative measurements were based on the axes of the medial and lateral humeral epicondyles, which may lead to differences in measurement with the axis of the forearm.

Analysis of the postoperative implant placement position and clinical evaluation in the present study showed a correlation between RS and postoperative ER. Biomechanical studies have reported that insertion within 0-20° maximizes IR and an increase in retroversion increases the ROM of ER.^4^^,^^10^^,^^13^^,^^18^ Furthermore, anteversion installation has been reported to decrease the ROM of ER.^29^ In contrast, Rhee et al compared 0° and 20° RS with a conventional guide and reported no significant differences in daily activities except higher IR activity scores at 0°.^26^ Similarly, De Boer et al compared 0° and 20° RS and reported no significant differences in the ROM of IR and ER and functional outcome scores. ^5^ Oh et al also reported that ROM of the ER and IR, functional score, and VAS were significantly better in the group with RS set to the preoperative HR and the group fixed at 20° than in those set to patient-specific values. However, a study reported that a subanalysis with the RS set at > 20° and within 0-20° revealed no significant differences.^24^

However, these studies set guided angles and did not measure postoperative RS. In a study measuring postoperative RS in 3D, Jang et al reported that RS correlated with ROM of flexion and abduction, and muscle strength of IR at a follow-up period of more than one year after surgery.^17^ In the present study, the postoperative RS correlated with the ROM of ER at 2 years postoperatively, and in a subanalysis, the Constant–Murley score was significantly higher in group 1 than in group 2. The results on ROM of ER supported those of a biomechanical study and suggested that placement between 0° and 20° may be useful in improving clinical outcomes. To improve the accuracy of stem positioning, it is necessary to develop surgical-assistive technologies, such as patient-specific implant and navigation, in the future.

Inferior inclination of the glenoid is important to avoid early **c**omponent loosening and scapular notching. Biomechanical studies have shown that GI is associated with a better ROM for adduction and ER.^22^^,^^25^ However, there are reports that GV and GI are not associated with the actual ROM or clinical outcomes.^30^ In congruence, the present study found no significant associations. Regarding GO, biomechanical studies have shown that glenoid lateralization improves ROM in adduction and abduction.^11^^,^^33^ Lädermann et al also reported an improved ROM in all directions.^21^ Conversely, there are reports that glenoid lateralization is not associated with actual ROM and clinical outcomes.^20^ GO, in this study, measured the distance from the glenoid fossa to the greater tuberosity of the humerus; however, the osteotomy line and polyethylene insert were constant, suggesting a significant influence of glenoid lateralization. However, there was no correlation between clinical outcomes and ROM.

The limitations of this study included its retrospective design and the small number of cases. However, the association between RS and postoperative ER had a moderate effect size (0.6) in the post hoc power analysis test. In addition, the implant used was a single model. Therefore, it may not be appropriate for other models. The results were also evaluated at 2 years postoperatively, and clinical results over a longer period are needed. Furthermore, measurement errors may have occurred because the basic axes in this study were the medial and lateral humeral epicondyles, whereas the forearm was the index of intraoperative RS. However, with a standard error of 11.9°, we consider the error from the target value to be significant. Finally, as this RS procedure was performed by a single surgeon, the learning curve and conditions may be different for other surgeons. Nevertheless, we believe that surgical technique was standardized as the surgeon in this study had extensive prior experience in performing surgery.

## Conclusion

Placement of the RS using a conventional guide varied according to the target position. RS correlated with postoperative ER, and RS within 0°-20° significantly improved clinical outcomes. These results suggest that accurate placement of the RS may improve clinical outcomes, and the development of devices for accurate placement is needed.

## Disclaimers

Funding: No funding was disclosed by the authors.

Conflict of interest statement: The authors, their immediate families, and any research foundations with which they are affiliated have not received any financial payments or other benefits from any commercial entity related to the subject of this article.
